# Emotional Distress in Cancer Patients During the First Wave of the COVID-19 Pandemic

**DOI:** 10.3389/fpsyg.2021.755965

**Published:** 2021-11-05

**Authors:** Patricia Toquero, Carmen Blanco Fernández, María Pilar López Martí, Berta Hernández Marín, E. Beatriz Vera Cea, Ana Garrido García, Elena Méndez Carrascosa, Dulce Bañón Torres, Olga Donnay Candil, Ana Isabel Ballesteros García, José Miguel Sánchez-Torres, Pablo Costas Rojo, Rebeca Mondéjar, Ramon Colomer, Nuria Romero-Laorden

**Affiliations:** ^1^Department of Medical Oncology, Hospital Universitario de La Princesa, Madrid, Spain; ^2^Spanish Association Against Cancer (Asociación Española Contra el Cáncer, or “AECC”), Madrid, Spain; ^3^Department of Medical Oncology, Hospital General Nuestra Sra. Del Prado, Talavera de la Reina, Spain

**Keywords:** emotional distress, cancer, HADS – Hospital Anxiety and Depression Scale, COVID-19, anxiety, depression

## Abstract

**Background:** The COVID-19 pandemic has caused mental health problems worldwide. The psychopathological implications of COVID-19 in cancer patients have rarely been addressed. Considering the increased vulnerability of oncology patients, this issue needs to be addressed to improve the long-term mental health status of these patients.

**Methods:** We conducted a prospective study in outpatients under active cancer treatment during the first wave of the COVID-19 pandemic. A semi-structured 24-question survey was designed to measure baseline sociodemographic, psychosocial and COVID-19 exposure characteristics. The Hospital Anxiety and Depression Scale was used to measure psychological symptoms. A descriptive and analytical univariate analysis of the variables studied was performed. We used the *Z*-score to compare different populations (experimental and historical control cohort).

**Results:** 104 patients were included, the majority of which were women (64.4%), were above 65 years of age (57.7%), had either lung and breast cancer (56.7%), had advanced disease (64%) and were undergoing chemotherapy (63.5%). 51% of them expressed greater fear of cancer than of COVID-19 infection or both.

In relation to HADS, 52.8% of emotional distress, 42.3% of anxiety and 58.6% of depression rates were detected. The main factors related with higher rates of psychological symptomatology were history of previous psychotropic drug consumption and the adoption of additional infection prevention measures because they considered themselves at risk of severe COVID-19 infection (*p* = 0.008; *p* = 0.003 for emotional distress, *p* = 0.026; *p* = 0.004 for anxiety, and *p* = 0.013; *p* = 0.008 for depression). Tumor type, stage, oncologic treatment or rescheduling of cancer treatments were not related to higher levels of psychological symptomatology.

Comparison of our results with another population of similar characteristics was not significant (*Z* score = −1.88; *p* = 0.060).

**Conclusions:** We detected high rates of emotional distress during the first wave of the COVID-19 pandemic among cancer patients in active treatment (52.8%). This was higher and clinically relevant than observed in a comparable population (42.5%), although not significant. Cancer itself is the main factor of concern for cancer patients, above and beyond the emotional distress generated by COVID-19 pandemic.

## Introduction

Since the COVID-19 pandemic showed up, the therapeutic and prognostic impact of COVID-19 infection on cancer patients has been evaluated (Curigliano et al., 2020; Liang et al., 2020; Sociedad Española de Oncología Médica [SEOM], 2020; Trilla, 2020; World Health Organization [WHO], 2020), suggesting a higher mortality and higher risk of developing complications (Saini et al., 2020). The greater physical vulnerability of cancer patients infected with Sars-Cov-2 seems to be related to the state of immunosuppression associated, caused by the tumor process itself and by the administration of antineoplastic treatments, the presence of comorbidities, and the need to regularly visit a hospital (Miyashita et al., 2020; Petrova et al., 2020; Zhang et al., 2020). At Hospital Universitario de La Princesa we recently published a mortality rate for COVID-19 disease in cancer patients of 33% (Toquero Diez et al., 2020), similar to that reported in other national and international centers (Lee et al., 2020).

Most studies in cancer patients infected with COVID-19 have focused on the clinical course and treatment of the infection (Wang and Zhang, 2020; Zhang et al., 2020). However, we do not have studies addressing the emotional impact that the first wave of the pandemic has had on oncology patients who were under active treatment, and who, therefore, regularly attended a hospital center during Spain’s strictest months of lockdown and worst epidemiological situation of the pandemic.

Psychopathological disorders (emotional distress, anxiety, and depression) in cancer patients are frequent and exceed those observed in the general population (Pitman et al., 2018). Moreover, they differ widely according to the stage of the oncological disease, type of patient (ambulatory or hospitalized), treatment received, and various social, psychological, and physical factors (Hernández et al., 2013). Several studies have been carried out in the oncologic population evaluating psychopathological symptomatology (Moncayo et al., 2008; Hernández and Antonio Cruzado, 2013; Hernández et al., 2013). Despite differences in the development and interpretation of the results, all have shown the importance of the psychological approach in the comprehensive treatment of cancer patients, especially in the most vulnerable. This has also been demonstrated in the context of other epidemics. Studies evaluating the emotional impact of the SARS epidemic in 2004 or Ebola in 2014 have shown a worsening in psychological symptomatology rates in the global population, and to a greater extent, among the most vulnerable groups such as cancer patients (Brooks et al., 2020). Therefore, the COVID-19 pandemic is likely to magnify mental health problems in cancer patients (Agua et al., 2020; Swainston et al., 2020; Wang et al., 2020).

The studies published to date about the emotional impact caused by the COVID-19 pandemic in oncology patients are scarce, and the different studies have important methodological differences, mainly due to the use of different scales for measuring psychopathological symptomatology (HADS, CES-D, GSDS, etc.)—some of which are not clearly validated in this type of population—and also because they used different cut-off points. The studied populations are heterogeneous (patients with cancer under active treatment or follow-up, long survivors, different types of tumors or tumor stage, etc.), which make it difficult to compare or extrapolate conclusions (Agua et al., 2020; Letaief-Ksontini et al., 2020; Miaskowski et al., 2020; Swainston et al., 2020; Wang et al., 2020). In this regard, rates of depression and anxiety in cancer patients reported during the COVID-19 pandemic vary widely between 11.8–35.3 and 9.9–69.2%, respectively (Agua et al., 2020; Letaief-Ksontini et al., 2020; Lou et al., 2020; van de Poll-Franse et al., 2020; Wang et al., 2020; Gultekin et al., 2021; Mogami et al., 2021).

In our knowledge, this is the first prospective study to evaluate the emotional and social implications of the COVID-19 pandemic in cancer outpatients who were on active treatment exclusively, and who regularly attended a hospital center during the first and arguably most impactful wave of the COVID-19 pandemic. Thus, the main objective of this study is to explore the level of emotional distress and anxious and/or depressive symptomatology of oncology patients during the first wave of the COVID-19 pandemic, and subsequently, to study the relationship between psychological symptomatology of these patients and a range of sociodemographic, clinical, economic, and spiritual factors.

## Materials and Methods

### Study Design and Patients

We conducted a prospective descriptive correlational study, including (a) adult patients (b) with a diagnosis of solid tumor (c) who were undergoing active oncologic treatment, and who (d) regularly attended to the oncohematologic Day Hospital (DH) at Hospital Universitario de La Princesa (HULP) (e) between May 18th and June 5th, 2020. All patients who were treated in the medical oncology service during this period, and consequently attended face-to-face consultations, were offered the possibility of participating in this study voluntarily. No payment was received for their contribution to this study.

### Ethical Approval Statement

The study was developed in accordance with the fundamental principles of Good Clinical Practice established in the Declaration of Helsinki. It was approved by the Drug Research Ethical Committee (CEIm) of the *Hospital Universitario de La Princesa* (*registration number “4122,” approved 22-05-20, CEIm 12/20*). All patients signed an informed consent (IC) form prior to their participation in the study, and the questionnaire responses were anonymized.

### Data Collection

Data collection was performed using two types of questionnaires. A 24-question semi-structured questionnaire was designed to measure clinical and sociodemographic variables and the exposure level to COVID-19. This questionnaire was designed considering possible risk factors related to elevated rates of emotional distress in cancer patients and based on previous clinical evidence (Agua et al., 2020; Letaief-Ksontini et al., 2020; Lou et al., 2020; Swainston et al., 2020; van de Poll-Franse et al., 2020; Wang et al., 2020; Gultekin et al., 2021; Mogami et al., 2021). The HADS (Hospital Anxiety and Depression Scale) questionnaire was used to measure variables related to psychological symptomatology. Both surveys were completed individually by the patients during their stay at the oncohematologic DH, only the items related to oncologic disease were recorded by the researchers to ensure reliability. A complete copy of the questionnaire (general questionnaire and HADS) can be found in the Supplementary Material.

(A) General Questionnaire:

It consisted of 24 questions including the variables below:

• Sociodemographic and comorbidity: age, sex (male/female), marital status (single/married/divorced/widowed), educational level (high school graduate/University education/no education), etc.
• Related to oncologic disease: tumor type, tumor stage (localized/metastatic), type of treatment administered (chemotherapy/immunotherapy/other), etc.
• COVID-19-related: presence of active COVID-19 infection (yes/no), living with COVID-19-positive patients (yes/no), number of visits to HULP during the first wave of the pandemic, level of information received about COVID-19 (a lot to some/little to none), COVID-19-caused death of family member or close contact (yes/no), etc.

(B) HADS (Hospital Anxiety and Depression Scale):

The HADS scale is a well-known emotional distress self-report questionnaire, and one of the most frequently used in oncology. It was selected as the optimum method for measuring psychological symptomatology in this study. This scale has been widely validated and used as a reliable self-assessment instrument for screening for depression and anxiety in the hospital setting in the general population (Herrero et al., 2003), and also in cancer patients (Snaith, 2003; Grassi et al., 2004; Moncayo et al., 2008; Mitchell et al., 2010). Unlike other screening methods, it does not include references to somatic symptoms derived from emotional stress, such as weight loss or insomnia, which could be caused directly by the disease itself or related to the oncological treatment (Mitchell et al., 2010). It is composed of two subscales (HADS-A: anxiety, and HADS-D: depression) of seven items each, answered using a 4-point Likert scale (from 0 to 3) (Hernández et al., 2013). The scores for each subscale are 0–21 and 0–42 for the full scale. Both subscales are broadly correlated with each other [Cronbach’s alpha, a measure of scale reliability, for HADS-A varies between 0.68 and 0.93 (mean 0.83) and for HADS-D between 0.67 and 0.90 (mean 0.82) (Bjelland et al., 2002)].
According to the available evidence for cancer outpatient population, a cut-off point of 10 (0–9 vs. 10–42) was used for emotional distress screening, 7 for anxiety screening (0–6 vs. 7–21) and 4 (0–3 vs. 4–21) for depression, both with a sensitivity and specificity of more than 70–80% (Moncayo et al., 2008). Higher scores above the established cut-off point were associated with higher rates of psychopathological symptomatology.

### Statistical Analysis

An initial descriptive analysis of the sociodemographic, clinicopathological, and psychological variables of the surveyed patients was performed. For quantitative variables, measures of central tendency and dispersion were used (arithmetic mean and standard deviation, median and interquartile range). For qualitative variables absolute and relative frequencies in percent. The possible association between the principal variables (emotional distress, anxiety, and depression) and the secondary sociodemographic variables collected was studied using Pearson’s chi-square (χ^2^) for qualitative variables (Fisher’s exact test, if both variables were dichotomous) and the Student’s *t*-test for quantitative variables. All p tests were two-tailed (two tails) and a value of <0.05 was considered statistically significant due to exploratory nature of the study. The IBM SPSS Statistics 25 program was used for the statistical analysis.

To compare results of psychopathological symptomatology in a different population with similar characteristics we used the “*Z* score” (Population A; the experimental cohort, and population B; the control group – historical control subject). This value enables us to compare two scores that are from different samples (which may have different means and standard deviations). Questionaries with incomplete data were excluded from the final analysis (*N* = 26).

#### Sample Size

As an exploratory study, there was not enough previous evidence available to estimate an adequate sample size. The prevalence of psychological disorders in cancer patients reported is higher compared with the general population (Hernández and Antonio Cruzado, 2013), however, the variability among studies makes it difficult to know the real prevalence of these types of disorders. In our study, due to the impossibility to guarantee a correct randomization of patients during the first wave of the pandemic, we decided to use a historical cohort like control group. In this sense, for the comparison of our results we used those reported in the study by Moncayo et al. (2008), which used a methodology and patient sample very similar to ours, considering a prevalence of emotional distress of 42.5% (Moncayo et al., 2008) in the reference group. Accepting an alpha risk of 0.05 and a beta risk of 0.2 in bilateral contrast, we estimated the need for a minimum of 95 subjects to detect a difference equal to or greater than 15%. A 10% of losses rate was also considered.

## Results

### Baseline Characteristics (Sociodemographic, Psychosocial and Clinical Characteristics Related to Oncologic Disease)

The main baseline characteristics of the studied population (*N* = 104) are shown in Table 1. The mean age was 62 years (29–87) 64.4% were women, 53.8% were married, and 57.7% had a high school diploma. Ten patients (9.6%) were smokers and 57.7% occasionally consumed alcohol. Seven patients (6.7%) had a previous diagnosis of major psychiatric disorder, although 70% affirmed that they were treated with benzodiazepines or antidepressants at the time of the study.

**TABLE 1:** Baseline characteristics of the surveyed population (*n* = 104).

**Sociodemographic characteristics**	**Patients under active treatment at HULP**
**Age**	
• Under 65 years of age	60 (57.7%)
• Over 65 years of age	44 (42.3%)
**Gender**	
• Female	67 (64.4%)
• Male	37 (35.6%)
**Marital status**	
• Married	56 (53.8%)
• Single	28 (26.9%)
• Divorced/separated	12 (11.5%)
• Widowed	8 (7.7%)
**Level of education**	
• University education	40 (38.5%)
• High school graduate	60 (57.7%)
• None	4 (3.8%)

**Psychosocial characteristic**	

**Psychiatric disease (DSM-V)**	
• No	95 (91.3%)
• Yes	7 (6.7%)
**Used of psychotropic drugs (BZD/AD)**	
• No	72 (70.5%)
• Yes	30 (29.4%)
**Smoker**	
• No	93 (89.4%)
• Yes	10 (9.6%)
Alcohol consumption *1	
• None	39 (37.5%)
• Occasional (1–2 alcoholic beverages/week)	60 (57.7%)
• Frequent (almost every day)	5 (4.8%)

**Clinical characteristics (oncologic disease)**	

Type of tumor *2	
• Lung cancer	31 (29.8%)
• Breast cancer	28 (26.9%)
• Colorectal cancer	12 (11.5%)
**Tumor stage**	
• Metastatic	66 (63.5%)
• Localized	38 (36.5%)
**Anti-cancer treatment**	
• Chemotherapy	66 (63.5%)
• Immunotherapy	21 (20.2%)
• Other	15 (14.4%)

**Number of visits oncohematologic DH during the first wave of the COVID-19 Pandemic**	

• Median	5 (1–30)
• Up to 4	37 (35.6%)
• More (≥) than 5	67 (64.4%)
**Rescheduling of oncologic treatment:**	
• No	76 (73%)
• Yes	28 (27%)

*^1^None had an excessive consumption.

*^2^Others type of tumor: digestive cancer (non-colorectal) 16 (15.4%), Genitourinary.

The most frequently diagnosed tumor was lung cancer (29.8%), followed by breast cancer (26.9%). Most of the patients surveyed (63.5%) had metastatic cancer, with 63.5% undergoing chemotherapy. 51% of the patients expressed greater concern about their tumor compared with the risk of COVID-19 infection, 18% had COVID-19 as main concern, and 31% were equally concerned about both.

### Characteristics Related to Exposure to COVID-19

The mean number of visits to HULP during the first wave of the COVID-19 pandemic was 5 (1–30), and 27.9% of the patients visited the hospital more than 10 times. 94% of the patients had not been diagnosed of COVID-19. Six percent (6%) lived with COVID-19-positive patients. The financial situation of 24% patients worsened since the start of the pandemic. 40% of the patients used religion as a method of reflection and relief, and 10% resorted to different types of relaxation therapies (Table 2).

**TABLE 2:** Baseline characteristics of the surveyed population (*n* = 104).

**Characteristics related to exposure to COVID-19**	**Patients under active treatment at HULP**
**Previous COVID-19 infection:**	
• No (98)	98 (94.2%)
• Yes (6)	6 (5.8%)
**Living with a COVID-19-positive patient:**	
• No (98)	98 (94.2%)
• Yes (6)	6 (5.8%)
**Information level on COVID-19:**	
• A lot/considerable (77)	77 (74%)
• Little/none (27)	27 (26%)
**Increased concern for/fear of:**	
• Tumor (53)	53 (51%)
• COVID-19 (19)	19 (18.2%)
• Both equally (32)	32 (30.8%)
**Death or admission of family member/close friend:**	
• No (68)	68 (65,4%)
• Yes (36)	36 (34.6%)
**Increased prevention of COVID-19 infection as an oncology patient:**	
• A lot/considerable (83)	83 (79.8%)
• Little/none (21)	21 (20.2%)
**Family/social support:**	
• A lot/considerable (98)	98 (94.2%)
• Little/none (6)	6 (5.8%)
**Worsened financial situation:**	
• Little/none (79)	79 (76%)
• A lot/considerable (25)	25 (24%)
**Support provided by religion:**	
• Little/none (62)	62 (60%)
• A lot/considerable (42)	42 (40%)
**Support provided by relaxing therapies:**	
• A lot/considerable (10)	94 (90.4%)
• Little/none (94)	10 (9.6%)
**Media exposure**	
• A lot/considerable (>2 times per day) (57)	57 (54.8%)
• Little/none (≤1 time per day) (47)	47 (54.2%)

### Psychopathological Symptomatology

The results obtained from the study of psychopathological symptomatology (emotional distress, anxiety, and depression) are presented in Tables 3, 4, according to the presence of patient baseline characteristics or COVID-19-related variables.

**TABLE 3:** Baseline characteristics (sociodemographic, comorbidity, and oncologic disease) and existence of emotional distress, anxiety, and depression (HADS scale).

**Variables**	**HADS-T**	**HADS-A**	**HADS-D**	***p-*HADS-T**	***p-*HADS-A**	***p-*HADS-D**
	
	**Case**	**Case**	**Case**			

**TOTAL (104 patients)**	**55 (52.8%)**	**44 (42.3%)**	**61 (58.6%)**			
**Age:**						
• <65 years old (60)	36 (60%)	29 (48.3%)	37 (61.7%)	NS	NS	NS
• ≥65 years old (44)	19 (43.2%)	15 (34.1%)	24 (54.5%)			
**Gender:**						
• Female (67)	38 (56.7%)	31 (46.3%)	42 (62.7%)	NS	NS	NS
• Male (37)	17 (45.9%)	13 (35.1%)	19 (51.4%)			
**Marital status:**						
• Married (56)	31 (55.4%)	23 (41.1%)	32 (57.1%)	NS	NS	NS
• Single (28)	16 (57.1%)	13 (46.4%)	18 (64.3%)			
• Divorced (12)	4 (33.3%)	4 (33.3%)	6 (50%)			
• Widowed (8)	4 (50%4)	4 (50%)	5 (62.5%)			
**Level of education:**						
• University education (40)	1 7 (42.5%)	16 (40%)	18 (45%)	NS	NS	**0.032**
• High school graduated (60)	35 (58.3%)	25 (41.7%)	39 (65%)			
• None (4)	3 (75%)	3 (75%)	4 (100%)			
**Previous psychiatric illness:**						
• No (95)	47 (49.5%)	38 (40%)	53 (55.8%)	NS	NS	NS
• Yes (7)	6 (85.7%)	5 (71.4%)	6 (85.7%)			
**Previous used of psychotropic drugs (BZD/AD):**						
• No (72)	32 (44.4%)	26 (36.1%)	36 (50%)	**0.008**	**0.026**	**0.013**
• Yes (30)	22 (73.3%)	18 (60%)	23 (76.7%)			
**Smoker**						
• No (93)	50 (53.8%)	41 (44.1%)	54 (58.1%)	NS	NS	NS
• Yes (10)	4 (40%)	2 (20%)	6 (60%)			
**Alcohol consumption**						
• None (39)	24 (61.5%)	21 (53.8%)	25 (64.5%)	NS	NS	NS
• Occasional (1–2 alcoholic beverages/week) (60)	27 (45%)	21 (35%)	32 (53.3%)			
• Frequent (almost every day) (5)	4 (80%)	2 (40%)	4 (80%)			
**Type of cancer:**						
• Lung cancer (31)	18 (58.1%)	13 (41.9%)	19 (61.3%)	NS	NS	NS
• Breast cancer (28)	16 (57.1%)	14 (50%)	16 (57.1%)			
• Colorectal cancer (12)	5 (41.7%)	3 (25%)	5 (41.7%)			
**Tumor stage:**						
• Metastatic (66)	33 (50%)	27 (40.9%)	39 (59.1%)	NS	NS	NS
• Localized (38)	22 (57.9%)	17 (44.7%)	22 (57.9%)			
**Active treatment (during the COVID-19 pandemic):**						
• Chemotherapy (66)	35 (53%)	31 (47%)	38 (57.6%)	NS	NS	NS
• Immunotherapy (22)	9 (40.9%)	6 (27.3%)	13 (59.1%)			
• Other (16)	11 (68.8%)	7 (43.8%)	10 (62.5%)			
**Number of visits oncohematologic DH during the first wave of the COVID-19 pandemic**						
• Less than 5 visits (37)	21 (56,8%)	18 (48,6%)	22 (59.2%)	NS	NS	NS
• More (≥) than 5 visits (67)	34 (50.7%)	26 (38.8%)	39 (58.2%)			
**Rescheduling of oncologic treatment:**						
• No (76)	39 (51.3%)	34 (44.7%)	43 (56.6%)	NS	NS	NS
• Yes (28)	16 (57.1%)	10 (35.7%)	18 (64.3%)			

*Descriptive and univariate analysis using Chi-square distribution. HADS-T, Hospital Anxiety and Depression Scale-Combined subscales [as “case” we considered cutoff of 10 (0–9 vs. 10–42)]. HADS-A; Hospital Anxiety and Depression Scale-Anxiety subscale (as “case” we considered cutoff of 7 (0–6 vs. 7–21). HADS-D, Hospital Anxiety and Depression Scale-Depression subscale [as “case” we considered cutoff of 4 (0–3 vs. 4–21)]. BZD, benzodiazepines; AD, anti-depressants. NS, no significant (*p* < 0,05). The bold values represent statistical significance.*

**TABLE 4:** Variables related to exposure to COVID-19, and the existence of emotional distress, anxiety, and depression (HADS scale).

**Variables**	**HADS-T**	**HADS-A**	**HADS-D**	***p-*HADS-T**	***p-*HADS-A**	***p-*HADS-D**
	
	**Case** **(10–42)**	**Case** **(7–21)**	**Case** **(4–21)**			
**Previous COVID-19 infection:**						
• No (98)	51 (52%)	42 (42.9%)	57 (58.2%)	NS	NS	NS
• Yes (6)	4 (66.7%)	2 (33.3%)	4 (66.7%)			
**Living with a COVID-19-positive patient:**						
• No (98)	51 (51%)	42 (42.9%)	57 (58.2%)	NS	NS	NS
• Yes (6)	4 (66.7%)	2 (33.3%)	4 (66.7%)			
**Information level on COVID-19:**						
• A lot/considerable (77)	37 (48.1%)	32 (41.6%)	42 (54.5%)	NS	NS	NS
• Little/none (27)	18 (66.7%)	12 (44.4%)	19 (70.4%)			
**Increased concern for/fear of:**						
• Tumor (53)	24 (45.3%)	19 (35.8%)	29 (54.7%)	NS	NS	NS
• COVID-19 (19)	10 (52.6%)	8 (42.1%)	9 (47.4%)			
• Both equally (32)	21 (65.6%)	17 (53.1%)	23 (71.9%)			
**Death or admission of family member/close friend:**						
• Yes (36)	23 (63.9%)	18 (50%)	25 (69.4%)	NS	NS	NS
• No (68)	32 (47.1%)	26 (38.2%)	36 (52.9%)			
**Increased prevention of COVID-19 infection as an oncology patient:**						
• A lot/considerable (83)	50 (60.2%)	41 (49.4%)	54 (65.1%)	**0.003**	**0.004**	**0.008**
• Little/none (21)	5 (23.8%)	3 (14.3%)	7 (33.3%)			
**Family/social support:**						
• A lot/considerable (98)	51 (52%)	41 (41.8%)	56 (57.1%)	NS	NS	NS
• Little/none (6)	4 (66.7%)	3 (50%)	5 (83.3%)			
**Worsened financial situation:**						
• A lot/considerable (25)	20 (80%)	14 (56%)	21 (84%)	**0.002**	NS	**0.003**
• Little/none (79)	35 (44.3%)	30 (38%)	40 (50.6%)			
**Support provided by religion:**						
• A lot/considerable (42)	26 (61.9%)	22 (52.4%)	30 (71.4%)	NS	NS	**0.029**
• Little/none (62)	29 (46.8%)	22 (35.5%)	31 (50%)			
**Support provided by relaxing therapies:**						
• A lot/considerable (10)	7 (70%)	8 (80%)	7 (70%)	NS	**0.011**	NS
• Little/none (94)	48 (51.1%)	36 (38.3%)	54 (57.4%)			
**Media exposure**						
• A lot/considerable (>2 times per day) (57)	31 (54.4%)	29 (50.9%)	36 (63.2%)	NS	**0.05**	NS
• Little/none (≤1 time per day) (47)	24 (51.1%)	15 (31.9%)	25 (53.2%)			

*Descriptive and univariate analysis using Chi-square distribution. HADS-T, Hospital Anxiety and Depression Scale-Combined subscales [as “case” we considered cutoff of 10 (0–9 vs. 10–42)]. HADS-A, Hospital Anxiety and Depression Scale-Anxiety subscale [as “case” we considered cutoff of 7 (0–6 vs. 7–21)]. HADS-D, Hospital Anxiety and Depression Scale-Depression subscale [as “case” we considered cutoff of 4 (0–3 vs. 4–21)]. BZD, benzodiazepines; AD, anti-depressants. NS, no significant (*p* < 0,05). The bold values represent statistical significance.*

#### Hospital Anxiety and Depression Scale—Total/Emotional Distress

A 52.8% rate of emotional distress or global discomfort was observed.

Significantly higher rates of emotional distress were detected in patients who had previously consumed benzodiazepines or antidepressants (73.3% vs. 44.4%, *p* = 0.008), in those who maintained more prevention measures than recommended for COVID-19 infection because they considered themselves at higher risk (60.2% vs. 23.8%, *p* = 0.003) and in those whose economic situation had worsened during pandemic (80% vs. 44.3%, *p* = 0.002).

A non-significant trend was observed for the association between higher levels of emotional distress and being young (<65 years), having a low educational level, being single, and being female. Tumor type, tumor stage, and type of oncologic treatment administered were not significantly related to higher levels of emotional distress. The use of relaxing therapies, religion, or a high exposure to mass media, were also not significantly related to higher levels of global emotional disturbance.

#### Hospital Anxiety and Depression Scale—Anxiety Subscale

A 42.3% rate of anxiety was found.

Significantly higher rate of anxiety was observed in patients who had previously taken anxiolytics or antidepressants (60% vs. 36.1%, *p* = 0.026), those who adopted more measures to prevent contagion than recommended for general population because they considered themselves at higher risk for COVID-19(49.4% vs. 14.3%, *p* = 0.004), those who resorted to the use of relaxing therapies (80% vs. 38.3%, *p* = 0.011) and those who presented a higher level of exposure to the media (>2 times a day) (50.9% vs. 31.9%, *p* = 0.05).

Neither age, sex, educational level, or the presence of a confirmed psychiatric disease showed significant differences in the anxiety levels of the patients surveyed. Neither the characteristics of their oncologic disease (type of tumor, stage, type of oncologic treatment administered, or the rescheduling of treatment administration) significantly related to higher levels of anxiety. In patients with greater exposure to coronavirus at home, no statistically significant differences were observed in their anxiety levels (33.3% vs. 42.9%).

#### Hospital Anxiety and Depression Scale—Depression Subscale

A 58.6% rate of depression was detected.

Patients who showed significantly higher rates of depression were those with lower educational level (100% in patients with no education vs. 63% with elementary education and 45% with higher education, *p* = 0.032), those who admitted having previously consumed antidepressants or benzodiazepines (76.7% vs. 50%, *p* = 0.013), those who adopted more preventive measures than those recommended for the general population to avoid COVID-19 infection because they considered themselves at higher risk (65.1% vs. 33.3%, *p* = 0.008), those whose economic situation worsened due to the economic crisis caused by the pandemic (84% vs. 50.6%, *p* = 0.029) and those who relied on religion for support (71.4% vs. 50%, *p* = 0.029).

No significant differences were found in the levels of depression associated to patient age, sex, or marital status. Tumor stage, tumor type and oncologic treatment received were also not significantly related to the observed rates of depression.

### *Z*-Score

Comparison of our results with another population of similar characteristics as historical control group was not significant (*Z* score = −1.88; *p* = 0.060).

## Discussion

This project is the first study that addresses the emotional impact that the COVID-19 pandemic had on cancer patients who were under active treatment in the oncohematologic DH of the Hospital Universitario de La Princesa, during the first and harder stage of the COVID-19 pandemic (March-June 2020) in Madrid.

We detected an emotional distress rate of 52.8%, anxiety of 42.3%, and depression of 58.6%. Previous evidence on psychopathological symptomatology in outpatients with cancer is scarce. Furthermore, the presence of important methodological differences between studies makes the results difficult to compare (Carroll et al., 1993; Grassi et al., 2004; Annunziata et al., 2020). However, Moncayo et al. (2008) described the prevalence of emotional distress, anxiety and depression in the oncology population under active treatment using a methodology similar to ours, and therefore we use it as historical control to obtain an adequate comparator due to the impossibility to guarantee a correct randomization of patients during the first wave of the pandemic. The Gil-Moncayo study analyzed 400 ambulatory cancer patients, using the HADS scale as a screening method, and used the same cut-off points as in our study. They described a rate of emotional distress of 42.5% (HADS-T), anxiety of 36.5% (HADS-A), and depression of 45% (HADS-D) (Moncayo et al., 2008). Comparing these results with those at our center, we detect a clinical and relevant increase in the levels of emotional distress, anxiety, and depression in the outpatient oncology population in accordance with the non-pandemic situation and previously published literature (Moncayo et al., 2008), but these results were not statistically significant (*Z* score; *p* = 0.06). Despite a greater tendency, we consider that the increase detected was moderate, considering the profound psychological impact that COVID-19 pandemic has had on society worldwide, causing a significant increase in the rates of emotional distress, anxiety, and depression in the general population according to various studies (Pierce et al., 2020; Xiong et al., 2020).

Apart from our study, little evidence has analyzed the real impact from this pandemic on cancer patients and it may take several years to understand the real psychopathological implications in this population. Furthermore, there are important methodological discrepancies (different type of target patients, different scales for measuring psychopathological symptomatology, different endpoints, etc.) with heterogeneous results that are difficult to compare (Agua et al., 2020; Letaief-Ksontini et al., 2020; Lou et al., 2020; Swainston et al., 2020; Wang et al., 2020) (Table 5). Most of these studies carried out on cancer patients have detected an increase in perceived levels of anxiety, depression, and global discomfort, highlighting the importance of the emotional sphere in their comprehensive treatment (Agua et al., 2020; Letaief-Ksontini et al., 2020; Lou et al., 2020; Swainston et al., 2020; van de Poll-Franse et al., 2020; Wang et al., 2020; Gultekin et al., 2021; Mogami et al., 2021). In our study it is striking how the greatest concern of oncology patients continues to be their tumor—even more than the COVID-19 pandemic itself. Of 104 patients included in the study, 51% expressed greater fear of their tumor compared with coronavirus infection, which seems to indicate that the condition of being an oncology patient, exposed to end of life considerations, has a greater impact than contracting COVID-19 or the consequences of this disease. These data coincide with that published by Gultekin et al. (2021) in their study, where 58% of patients expressed greater fear of their tumor vs. COVID-19.

**TABLE 5:** Main studies evaluating emotional impact and COVID-19 in oncology patients using validated scales to measure psychopathological symptomatology compared with our results.

**Author**	**Country/region**	**N**	**Cancer patients’ characteristics**			**Scale**	**Results**
			**Type of patient**	**Treatment/follow-up**	**Type of tumor**		
Gultekin et al., 2021	EU	1251	Ambulatory patients	Scheduled for surgery (without oncologic treatment yet). Under active treatment (46%). Under follow-up.	Gynecological cancers in any stage	Online survey HADS; HADS-T not reported. ≥11; “significant case” of psychological morbidity (abnormal) 8–10; “borderline case” 0–7; “normality” (healthy individuals)	HADS-A (≥ 11): 35.3% HADS-A (8–10): 24.1% HADS-D (≥ 11): 30.6% HADS-D (8–10): 20.6%
Letaief-Ksontini et al., 2020	Turkey	91	NR	NR	NR	HADS	HADS-A: 29.7% HADS-D: 69.2%
Wang et al., 2020	China	6213	Ambulatory patients	NR	All types of tumors	Online survey Anxiety; GAD-7 Depression: PHQ-9	Anxiety rate: 17.7% Depression rate: 23.4%
Agua et al., 2020	Spain	2293	Ambulatory patients	Active treatment (50%). Follow up.	All types of tumors	Online survey Kessler Psychological Distress Scale K-6	Psychological distress rate: 34.2%
Lou et al., 2020	United States	543	Ambulatory patients	Oncology (55.4%)* and non-oncology patients (44.6%). *Active treatment (25.6% of the oncologic patients included)	All types of tumors	Online survey Anxiety; GAD-7 Depression: PHQ-8	^1^ Anxiety rate: 26% ^1^ Depression rate: 26.7%
van de Poll-Franse et al., 2020	Denmark	4094	Ambulatory patients	Oncology (4094)* and non-oncology patients (977). *Active treatment (27%)	All types of tumors	HADS	HADS-A: 11.8% (v. 11.2% in non-oncology patients) HADS-D: 9.9% (v. 12.2%)
Mogami et al., 2021	Japan	34	Ambulatory patients	Active treatment (65%). Follow up.	Gynecological cancers in any stage	HADS: HADS-A; ≥ 8 HADS-D; ≥ 8	Anxiety rate: 33% Depression rate: 43%
Toquero Diez et al., 2020 *(Psico-Covid)*	Spain	104	Ambulatory patients	Active treatment (100%).	All types of tumors	Staff-supervised HADS: HADS-T; ≥10 HADS-A; ≥7 HADS-D; ≥4	HADS-T: 52.8% HADS-A: 42.3% HADS-D: 58.6%

*EU, European Union; USA, United States of America; NR, Not reported; HADS, hospital anxiety and depression scale; HADS-T, hospital anxiety and depression scale—total/emotional distress; HADS-A, hospital anxiety and depression scale—anxiety subscale; HADS-D, hospital anxiety and depression scale—depression subscale; O-HDH, onco-hematologic day hospital; GAD-7, 7-item generalized anxiety disorder; PHQ-8/PHQ-9, 8-9 item patient health questionnaire; GHQ-12, 12-item general health questionnaire. ^1^Results only for oncology patients in active treatment.*

The observed rates of emotional distress, anxiety, and depression, which are close to pre-pandemic levels, may be related to several factors: (a) the time at which the study was conducted—late May and early June 2020, when the pandemic had already entered a de-escalation phase and care pressure had decreased significantly in Madrid so that the perception of fear had been reduced in the population; (b) the need for patients to visit a hospital center regularly to receive treatment, allowing them to have closer contact with health personnel (doctors, nurses, and team of psychologist of AECC), and to resolve their questions and concerns about the COVID-19 pandemic with medical staff directly. Studies in non-oncologic populations without regular contact with hospital staff have shown higher levels of psychopathological symptomatology (Parrado-González and León-Jariego, 2020; Talevi et al., 2020).

The factors significantly related to higher rates of emotional distress, anxiety, and depression were previous consumption of psychotropic drugs and the adoption of greater prevention measures than those recommended for general population to avoid the infection because they considered at higher risk of developing more severe complications by the COVID-19. A worsening of patient’s financial situation during the pandemic was associated with greater distress and depression, the use of alternative therapies and Religion with greater anxiety and depression, lower educational level with greater depression, and frequent exposure to the media with a higher level of anxiety. This data is consistent with that published in other studies, such as that of the Spanish Association Against Cancer (AECC) (Agua et al., 2020) or Wang et al. (2020), where a worsening financial situation, frequent exposure to the media, and greater concern about COVID-19 infection were shown to be risk factors associated with higher rates of psychopathological symptomatology. This seems to demonstrate that, although the media is useful to increase the level of information in society, at other times it can transmit contradictory or excessive information (Bendau et al., 2020; Joshua Morganstein, 2020). These results are also consistent with those published by the AECC study, where up to 50% of patients who were unable to disconnect from the media presented high levels of emotional disturbance (Agua et al., 2020).

Although not significantly, younger patients (those under the age of 65) presented higher levels of psychological symptomatology, something which could be explained by the fact that younger patients are more shocked by the COVID-19 disease than older, according to what has been previously published (Linden et al., 2012; Kim et al., 2013). Similarly, being female, single, having a previous diagnosis of psychiatric illness, lack of information about COVID-19, and lack of social or family support during the pandemic were also factors related to higher rates of emotional distress, anxiety, and depression, although not significantly—results similar to those published by Wang et al. (2020) and Gultekin et al. (2021).

No significant differences were found in the rates of psychopathological symptomatology according to tumor type, oncologic stage, or treatment administered in either the subscales (anxiety or depression) or in the global emotional distress scale, suggesting that the emotional impact of the COVID-19 pandemic was similar for all cancer patients, regardless of the stage of their disease. This finding is similar to the results published by Gultekin et al. (2021). The rescheduling of treatments or the delay of any of the treatment cycles did not have a negative impact on the emotional sphere, data which agrees with that published by Letaief-Ksontini et al. (2020). However, other studies have shown varying results, as in the study by Swainston et al. (2020), carried out on a English population of women with breast cancer (any stage and any type of treatment), which showed that those women who experienced disruptions to their oncology services had higher levels of general anxiety, depression, and COVID-19 related emotional vulnerability (Swainston et al., 2020).

Among the limitations of this study, we should take into consideration some weak points which could be considered in future lines of research. In terms of methodology, the main limitations are the unicenter design and the lack of randomization to obtain an adequate control group to compare our results. Because the Pandemic was a worldwide event experienced by the entire population at the same time, it was not possible to ensure the randomization of patients. The evaluation method used also presents some considerations that are important to point out. Although the questionnaire applied (HADS) is a simple and intuitive tool that has shown adequate psychometric properties to evaluate anxiety, depression and emotional distress symptomatology in the general population (Herrero et al., 2003), also in oncologic patients (Snaith, 2003; Grassi et al., 2004; Moncayo et al., 2008; Mitchell et al., 2010; Chapman et al., 2020; Swainston et al., 2020) it is only self-report questionnaire and did not evaluate other psychological and personality aspects that might play a role in psychological distress of cancer patients. It would therefore be advisable to include other measures related to this distinction in future research on cancer patients, giving special importance to face-to-face interviews with the patient as well as other negative consequences of the pandemic on oncology patients, such as the job insecurity created by the COVID-19 outbreak in oncology patients by not being able to attend their usual work because of their increased vulnerability, which could increase the risk of experiencing affective disorders (anxiety and depression) and worse cognitive function in cancer patients (Chapman et al., 2020). Finally, levels of emotional distress, anxiety, and depression may have varied throughout the pandemic, whereas our study was conducted only during the first wave, it might be interesting to replicate this study in the current pandemic situation.

The results of this study show the importance of increasing the accessibility of mental health services to cancer patients. It is urgent to focus the efforts and economic resources of future governments on guaranteeing adequate care for the psychological consequences of the COVID-19 pandemic, especially in the most vulnerable population, such as oncology patients (Chapman et al., 2020). Future research should continue to monitor the long-term effects of COVID-19 on the psychological health of cancer patients (Swainston et al., 2020).

## Conclusion

The COVID-19 pandemic has been a game changer in healthcare worldwide. Oncology patients are an at-risk population that needs special attention and care, and further studies are needed to help us improve and optimize the treatment of this infection in oncology patients. In our study, high rates of emotional distress have been detected during the first wave of the COVID-19 pandemic among cancer patients in active treatment, this was higher and clinically relevant than observed in a comparable population (42.5%), although not significant. Although cancer continues to be the main fear of oncology patients, it is also essential to know the impact of COVID-19 pandemic on the emotional sphere in order to ensure a holistic approach for these patients, and to increase efforts to make mental health services more accessible to cancer patients.

## Data Availability Statement

The datasets presented in this article are not readily available because due to privacy or ethical restrictions. Requests to access the datasets should be directed to PT, kiku.toquero@gmail.com.

## Ethics Statement

The studies involving human participants were reviewed and approved by Drug Research Ethical Committee (CEIm) of the Hospital Universitario de La Princesa (registration number “4122,” approved 22-05-20, CEIm 12/20). The patients/participants provided their written informed consent to participate in this study.

## Author Contributions

PT, CB, ML, BH, RC, and NR-L: conception and design. PT, CB, ML, BH, EBV, AGG, EM, OD, AB, JS-T, PC, RM, RC, and NR-L: provision of study material or patients. PT, ML, BH, EBV, AGG, EM, OD, AB, JS-T, PC, RM, RC, and NR-L: collection and/or assembly of data. PT, CB, ML, RC, and NR-L: data analysis and interpretation and manuscript writing. PT, CB, ML, BH, EBV, AGG, EM, OD, AB, JS-T, PC, RM, RC, and NR-L: final approval of manuscript. All authors contributed to the article and approved the submitted version.

## Conflict of Interest

The authors declare that the research was conducted in the absence of any commercial or financial relationships that could be construed as a potential conflict of interest.

## Publisher’s Note

All claims expressed in this article are solely those of the authors and do not necessarily represent those of their affiliated organizations, or those of the publisher, the editors and the reviewers. Any product that may be evaluated in this article, or claim that may be made by its manufacturer, is not guaranteed or endorsed by the publisher.

## References

[B1] Agua C. Y. Gázquez D. D. H. Sánchez B. F. Pérez E. B. (2020). *COVID-19 Emergencia en Cáncer. Diagnóstico Del Impacto Emocional Experimentado Por Las Personas Afectadas Por Cáncer Durante La Crisis Del COVID-19.* Available online at: https://www.aecc (accessed May 30, 2020).

[B2] Annunziata M. A. Muzzatti B. Bidoli E. Flaiban C. Bomben F. Piccinin M. (2020). Hospital Anxiety and Depression Scale (HADS) accuracy in cancer patients. *Supp. Care Cancer* 28 3921–3926. 10.1007/s00520-019-05244-8 31858249

[B3] Bendau A. Petzold M. B. Pyrkosch L. Mascarell Maricic L. Betzler F. Rogoll J. (2020). Associations between COVID-19 related media consumption and symptoms of anxiety, depression and COVID-19 related fear in the general population in Germany. *Eur. Arch. Psychiatry Clin. Neurosci.* 271 283–291. 10.1007/s00406-020-01171-6 32691135 PMC7371788

[B4] Bjelland I. Dahl A. A. Haug T. T. Neckelmann D. (2002). The validity of the Hospital Anxiety and Depression Scale An updated literature review. *J. Psychosom Res.* 52 69–77. 10.1016/s0022-3999(01)00296-3 11832252

[B5] Brooks S. K. Webster R. K. Smith L. E. Woodland L. Wessely S. Greenberg N. (2020). The psychological impact of quarantine and how to reduce it: rapid review of the evidence. *Lancet* 395 912–920. 10.1016/S0140-6736(20)30460-8 32112714 PMC7158942

[B6] Carroll B. T. Kathol R. G. Noyes R. Jr. Wald T. G. Clamon G. H. (1993). Screening for depression and anxiety in cancer patients using the Hospital Anxiety and Depression Scale. *Gen. Hosp. Psychiatry* 15 69–74. 10.1016/0163-8343(93)90099-a 8472942

[B7] Chapman B. Swainston J. Grunfeld B. Derakhshan N. (2020). COVID-19 outbreak effects on job security and emotional functioning amongst women living with breast cancer. *Front. Psychol.* 10.3389/fpsyg.2020.582014 29:582014. 33192902 PMC7658194

[B8] Curigliano G. Banerjee S. Cervantes A. Garassino M. C. Garrido P. Girard N. (2020). Managing cancer patients during the COVID-19 pandemic: an ESMO multidisciplinary expert consensus. *Ann. Oncol.* 31 1320–1335. 10.1016/j.annonc.2020.07.010 32745693 PMC7836806

[B9] Grassi L. Travado L. Moncayo F. L. Sabato S. Rossi E. Sepos Group (2004). Psychosocial morbidity and its correlates in cancer patients of the Mediterranean area: findings from the Southern European Psycho-Oncology Study. *J. Affect. Disord.* 83 243–248. 10.1016/j.jad.2004.07.004 15555721

[B10] Gultekin M. Ak S. Ayhan A. Strojna A. Pletnev A. Fagotti A. (2021). Perspectives, fears and expectations of patients with gynaecological cancers during the COVID-19 pandemic: a Pan-European study of the European Network of Gynaecological Cancer Advocacy Groups (ENGAGe). *Cancer Med.* 10 208–219. 10.1002/cam4.3605 33205595 PMC7753798

[B11] Hernández M. Antonio Cruzado J. (2013). La atención psicológica a pacientes con cáncer: de la evaluación al tratamiento. *Clín. Salud.* 24 1–9. 10.5093/cl2013a1

[B12] Hernández M. Cruzado J. A. Prado C. Rodríguez E. Hernández C. González M. A. (2013). Salud mental y malestar emocional en pacientes con cáncer. *Psicooncología* 9 233–257. 10.5209/rev_PSIC.2013.v9.n2-3.40895

[B13] Herrero M. J. Blanch J. Peri J. M. De Pablo J. Pintor L. Bulbena A. (2003). A validation study of the hospital anxiety and depression scale (HADS) in a Spanish population. *Gen. Hosp. Psychiatry.* 25 277–283. 10.1016/s0163-8343(03)00043-4 12850660

[B14] Joshua Morganstein M. D. (2020). *Coronavirus and Mental Health: taking Care of Ourselves During Infectious Disease Outbreaks.* United States: American Psychological Association.

[B15] Kim J. H. Yoon S. Won W. Y. Lee C. Lee C. U. Song K. Y. (2013). Age-specific influences of emotional distress on performance status in cancer patients: effects of emotional distress on performance in cancer patients. *Psychooncology* 22 2220–2226. 10.1002/pon.3276 23526824

[B16] Lee L. Y. W. Cazier J.-B. Starkey T. Briggs S. E. W. Arnold R. Bisht V. (2020). COVID-19 prevalence and mortality in patients with cancer and the effect of primary tumour subtype and patient demographics: a prospective cohort study. *Lancet Oncol.* 21 1309–1316. 10.1016/S1470-2045(20)30442-3 32853557 PMC7444972

[B17] Letaief-Ksontini F. Zenzri Y. Yahyaoui Y. Gabsi A. Mokrani A. Meddeb K. (2020). Anxiety and depression in cancer patients during the COVID-19 pandemic: a single-centre study. *Ann. Oncol.* 31 S957–S958. 10.1016/j.annonc.2020.08.2056

[B18] Liang W. Guan W. Chen R. Wang W. Li J. Xu K. (2020). Cancer patients in SARS-CoV-2 infection: a nationwide analysis in China. *Lancet Oncol.* 21 335–337. 10.1016/S1470-2045(20)30096-6 32066541 PMC7159000

[B19] Linden W. Vodermaier A. MacKenzie R. Greig D. (2012). Anxiety and depression after cancer diagnosis: prevalence rates by cancer type, gender, and age. *J. Affect. Disord.* 141 343–351. 10.1016/j.jad.2012.03.025 22727334

[B20] Lou E. Teoh D. Brown K. Blaes A. Holtan S. G. Jewett P. (2020). Perspectives of cancer patients and their health during the COVID-19 pandemic. *PLoS One.* 15:e0241741. 10.1371/journal.pone.0241741 33125442 PMC7598454

[B21] Miaskowski C. Paul S. M. Snowberg K. Abbott M. Borno H. Chang S. (2020). Stress and Symptom Burden in Oncology Patients During the COVID-19 Pandemic. *J. Pain Symptom Manage.* 60 e25–e34. 10.1016/j.jpainsymman.2020.08.037 32889039 PMC7462969

[B22] Mitchell A. J. Meader N. Symonds P. (2010). Diagnostic validity of the Hospital Anxiety and Depression Scale (HADS) in cancer and palliative settings: a meta-analysis. *J. Affect. Disord.* 126 335–348. 10.1016/j.jad.2010.01.067 20207007

[B23] Miyashita H. Mikami T. Chopra N. Yamada T. Chernyavsky S. Rizk D. (2020). Do patients with cancer have a poorer prognosis of COVID-19? An experience in New York City. *Ann. Oncol.* 31 1088–1089. 10.1016/j.annonc.2020.04.006 32330541 PMC7172785

[B24] Mogami T. Onuma E. Aoki M. Kamiya N. Sukegawa A. Miyagi E. (2021). Increased anxiety and depression in patients with gynecologic cancers during the COVID-19 pandemic: a retrospective study from Japan. *Int. J. Gynaecol. Obstet.* 152 457–458. 10.1002/ijgo.13470 33155289 PMC9087569

[B25] Moncayo F. L. G. Requena G. C. Pérez F. J. Salamero M. Sánchez N. Sirgo A. (2008). Adaptación psicológica y prevalencia de trastornos mentales en pacientes con cáncer. *Med. Clín. Barc.* 130 90–92. 18261378 10.1157/13115354

[B26] Parrado-González A. León-Jariego J. C. (2020). COVID-19: factors associated with emotional distress and psychological morbidity in spanish population. *Rev. Esp. Salud. Publica* 8:e202006058. PMC11583131 32507849

[B27] Petrova D. Pérez-Gómez B. Pollán M. Sánchez M.-J. (2020). Implicaciones de la pandemia por COVID-19 sobre el cáncer en España. *Med. Clín.* 155 263–266. 10.1016/j.medcli.2020.04.011 32444323 PMC7205671

[B28] Pierce M. Hope H. Ford T. Ford P. T. Hatch P. S. Hotopf P. M. (2020). Mental health before and during the COVID-19 pandemic: a longitudinal probability sample survey of the UK population. *Lancet Psychiatry* 7 883–892. 10.1016/S2215-0366(20)30308-4 32707037 PMC7373389

[B29] Pitman A. Suleman S. Hyde N. Hodgkiss A. (2018). Depression and anxiety in patients with cancer. *BMJ* 25:k1415. 10.1136/bmj.k1415 29695476

[B30] Saini K. S. Tagliamento M. Lambertini M. McNally R. Romano M. Leone M. (2020). Mortality in patients with cancer and coronavirus disease 2019: a systematic review and pooled analysis of 52 studies. *Eur. J. Cancer* 139 43–50. 10.1016/j.ejca.2020.08.011 32971510 PMC7467090

[B31] Snaith R. P. (2003). The Hospital Anxiety And Depression Scale. *Health Qual. Life Outcomes* 1:29. 10.1186/1477-7525-1-29 12914662 PMC183845

[B32] Sociedad Española de Oncología Médica [SEOM] (2020). *Posicionamiento y recomendaciones SEOM sobre el cribado serol gico previo al inicio de una quimioterapia inmunosupresora durante la pandemia COVID-19 en pacientes asintom ticos sin infecci n COVID-19 conocida. Abril 2020*. Available online at: https://seom.org/otrosservicios/noticias/207966posicionamientorecomendaciones-seom-sobre-el-cribado-serologico-previo-al-inicio-deuna-quimioterapia-inmunosupresora-durante-la-pandemia-covid-19-pacientes-asintomaticos-sin-infeccion-covid-19-conocida (accessed April 28, 2020).

[B33] Swainston J. Chapman B. Grunfeld B. Derakhshan N. (2020). COVID-19 lockdown and its adverse impact on psychological health in breast cancer. *Front. Psychol.* 24:2033. 10.3389/fpsyg.2020.02033 32982846 PMC7476556

[B34] Talevi D. Socci V. Carai M. Carnaghi G. Faleri S. Trebbi E. (2020). Mental health outcomes of the COVID-19 pandemic. *Riv. Psichiatr.* 55 137–144. 10.1708/3382.33569 32489190

[B35] Toquero Diez P. Vera Cea B. Garrido Garcia A. Méndez Carrascosa E. R. Torres D. B. Bosch R. C. (2020). Cancer patients infected with COVID-19 at La Princesa Hospital: real world data study. *Ann. Oncol.* 31 S1023–S1023.

[B36] Trilla A. (2020). One world, one health: the novel coronavirus COVID-19 epidemic. *Med. Clin.* 154 175–177. 10.1016/j.medcli.2020.02.002 32289080 PMC7140244

[B37] van de Poll-Franse L. V. de R. B. H. Horevoorts N. J. E. May A. M. Vink G. R. Koopman M. (2020). Perceived Care and Well-being of Patients With Cancer and Matched Norm Participants in the COVID-19 Crisis: results of a Survey of Participants in the Dutch PROFILES Registry. *JAMA Oncol.* 7 279–284. 10.1001/jamaoncol.2020.6093 33237294 PMC7689559

[B38] Wang H. Zhang L. (2020). Risk of COVID-19 for patients with cancer. *Lancet Oncol.* 21:e181. 10.1016/S1470-2045(20)30149-2 32142621 PMC7129735

[B39] Wang Y. Duan Z. Ma Z. Mao Y. Li X. Wilson A. (2020). Epidemiology of mental health problems among patients with cancer during COVID-19 pandemic. *Transl. Psychiatry.* 10:263. 10.1038/s41398-020-00950-y 32737292 PMC7393344

[B40] World Health Organization [WHO] (2020). *COVID-19, Weekly Epidemiological and Operational Updates.* Switzerland: World Health Organization.

[B41] Xiong J. Lipsitz O. Nasri F. Lui L. M. W. Gill H. Phan L. (2020). Impact of COVID-19 pandemic on mental health in the general population: a systematic review. *J. Affect. Disord.* 277 55–64. 10.1016/j.jad.2020.08.001 32799105 PMC7413844

[B42] Zhang L. Zhu F. Xie L. Wang C. Wang J. Chen R. (2020). Clinical characteristics of COVID-19-infected cancer patients: a retrospective case study in three hospitals within Wuhan. China. *Ann. Oncol.* 31 894–901. 10.1016/j.annonc.2020.03.296 32224151 PMC7270947

