# Supplementary material for: Emotional Distress in Cancer Patients During the First Wave of the COVID-19 Pandemic
Source: Front Psychol. 2021 Nov 5;12:755965. doi: 10.3389/fpsyg.2021.755965 (PMC8602105; doi:10.3389/fpsyg.2021.755965)
Supplement: Supplementary file 1 [file Data_Sheet_1.docx]

**PHYSICIAN QUESTIONNAIRE**

***PSICO-COVID***: ***EMOTIONAL DISTRESS IN CANCER PATIENTS DURING THE FIRST WAVE OF THE COVID-19 PANDEMIC.***

IP: Patricia Toquero Díez

Collaborators: *Carmen Blanco Fernández, María del Pilar López Martí, Berta Hernández Marín, Beatriz Vera Cea, Ana Garrido García, Elena Méndez Carrascosa, Dulce Bañón Torres^,^ Olga Donnay Candil, Ana Isabel Ballesteros García, José Miguel Sánchez-Torres, Pablo Costas Rojo, Rebeca Mondéjar Solís, Ramon Colomer, Nuria Romero-Laorden.*

Medical Oncology Department, Hospital Universitario de La Princesa.

***PATIENT ID:***

| **Affiliation:** | | | |
| --- | --- | --- | --- |
| NHC paciente: |  | | |
| Date of Birth |  | | |
| **Clinical variables:** | | | |
| Type of tumor |  | | |
| Tumor stage | Localized | | Metastatic |
| Type of treatment administered Chemotherapy/Inmunotherapy/Target therapy/Others |  | | |
| Number of visits the patient has made to the day hospital from March 13, 2020 to the present. |  | | |
| Diagnosis of previous psychiatric illness | YES | NO | |
| Previous consumption of benzodiazepines/antidepressants | YES | NO | |

**PATIENT QUESTIONNAIRE**

***PSICO-COVID***: ***EMOTIONAL DISTRESS IN CANCER PATIENTS DURING THE FIRST WAVE OF THE COVID-19 PANDEMIC.***

IP: Patricia Toquero Díez

Collaborators: *Carmen Blanco Fernández, María del Pilar López Martí, Berta Hernández Marín, Beatriz Vera Cea, Ana Garrido García, Elena Méndez Carrascosa, Dulce Bañón Torres^,^ Olga Donnay Candil, Ana Isabel Ballesteros García, José Miguel Sánchez-Torres, Pablo Costas Rojo, Rebeca Mondéjar Solís, Ramon Colomer, Nuria Romero-Laorden.*

Medical Oncology Department, Hospital Universitario de La Princesa.

**PATIENT ID:**

| **CIRCLE OR "X" THE ANSWER:** |
| --- |
| Gender   1. Male b) Female |
| Date of Birth: |
| Marital Satuts   1. Single b) Married c) Divorced d) Widowed |
| Educational Level   1. Middle School Graduate 2. High School Graduate 3. University education 4. No education |
| Weight (kg): Heigh (cm): |
| Number of people living at home during the Lockdown: |
| Smoker   1. YES b) NO |
| Alcohol consumption   1. Occasionally: 1-2 alcoholic drinks/week)/frequently 2. Frequently: almost every day 3. Excessive: every day 4. None |

| **I. DIRECT EXPOSURE TO COVID-19** |
| --- |
| **CIRCLE OR "X" THE ANSWER:** |
| 1. Have you ever had coronavirus infection (with confirmatory diagnostic test)? 2. Yes b. No |
| 1. Have you been to the emergency department for suspected coronavirus infection?   a. Yes b. No |
| 1. Have you been admitted to a hospital for coronavirus infection?   a. Yes b. No |
| 1. Have you ever lived with someone infected with coronavirus?   a. Yes b. No |

| **II. COVID-19 EXPOSURE AND HOSPITAL VISITS** |
| --- |
| **CIRCLE OR "X" THE ANSWER:** |
| 1. Have you been afraid to go to the hospital during this time? 2. A lot b. Quite a lot c. Little d. Not at all |
| 1. Have you delayed or postponed any consultation for fear of contagion when going to the Hospital?   a. Yes b. No |
| 1. Has your oncologist delayed any of your oncology treatments?   a. Yes b. No |
| 1. If you answered yes to the previous question, has this fact increased your concern?   a. A lot b. Quite a lot c. Little d. Not at all |
| 1. Has your coping with the current situation improved after resolving doubts or concerns with the healthcare workers?   a. A lot b. Quite a lot c. Little d. Not at all |
| 1. Has the level of information about Covid-19 been enough? 2. A lot b. Quite a lot c. Little d. Not at all |
| 1. Did you consider the Hospital to be a safe environment during your visits?   a. A lot b. Quite a lot c. Little d. Not at all |
| 1. How have you regularly attended the Hospital?   a. Walking b. Public transportation c. Car |
| 1. What have you been more afraid of your tumor or the possibility of Covid-19 infection?   a. Tumor b. Covid-19 c. Both |
| 1. How often have you thought you might be infected with Covid-19?   a. Persistently, several times a day b. Often, several days a week  c. A little, once a week d. Almost never e. Never |
| 1. Has anyone close to you, family or friends, died and/or been hospitalized for Covid-19??   a. Yes b. No |

| **III. CONFINEMENT AND ALARM STATE** |
| --- |
| **CIRCLE OR "X" THE ANSWER:** |
| 1. Since when have you restricted your outdoor activities due to the Covid-19 pandemic?   a. **Before** the lockdown (March 13^th^-14 ^th^) b. **After** the lockdown (March 13^th^-14 ^th^) |
| 1. Have you adopted greater contagion preventions than recommended because of your status as an oncology patient?   a. A lot b. Quite a lot c. Little d. Not at all |
| 1. During lockdown, has your consumption of alcohol or tobacco increased?   a. A lot b. Quite a lot c. Little d. Not at all |
| 1. Have you had to take additional medication for depression or anxiety during lockdown?   a. A lot b. Quite a lot c. Little d. Not at all |

| **IV. SOCIO-ECONOMIC SUPPORT (I)** |
| --- |
| **CIRCLE OR "X" THE ANSWER:** |
| 1. Have you felt supported by your friends or family during this period? 2. A lot b. Quite a lot c. Little d. Not at all |
| 1. Has your economic situation worsened or it will worsen as a result of the current COVID-19 pandemic situation? 2. A lot b. Quite a lot c. Little d. Not at all |

| **IV. SOCIO-ECONOMIC SUPPORT (II)** |
| --- |
| **CIRCLE OR "X" THE ANSWER:** |
| 1. Has Religion (Faith, Church) been a support or comfort during this time? 2. A lot b. Quite a lot c. Little d. Not at all |
| 1. Have you practiced relaxing therapies such as meditation, mindfulness, yoga, etc. during this period? 2. A lot b. Quite a lot c. Little d. Not at all |

| **V. MEDIA EXPOSURE** |
| --- |
| **CIRCLE OR "X" THE ANSWER:** |
| 1. How much exposure have you had to the information given by the mass media (news, internet, radio, etc.) about the COVID-19 pandemic?? 2. A lot: internet searches (twitter, google, blogs...) several times a day (more than 3 times a day), every day. Watching the news at least 2 times a day or more. 3. Quite a lot: internet searches every day 1-2 times a day, watching the news 1 or 2 times a day. 4. Little: occasional internet searches (not daily), watching the news once a day. 5. None: I have hardly ever searched the internet for information, I do not watch the news daily. |

| **VI. PSYCHOLOGICAL SYMPTOMATOLOGY - HADS** |
| --- |
| **CIRCLE OR "X" THE ANSWER:** |
| 25**. A1** I feel tense or “wound up”:  3. Most of the time 2. A lot of the time 1. From time to time, occasionally 0. Not at all |
| 26**. A2** I get short of frightened feeling as if something awful is about to happen:  3. Very definitely and quite badly 2. Yes, but not too badly  1. A Little, but it doesn`t worry me 0. Not at all |
| 27. **A3** Worrying thoughts go through my mind:  3. A great deal of the time 2. A lot of the time 1. From time to time, but not too often  0. Not at all |
| 1. **A4** I can sit at ease and feel relaxed:   0. Definitely 1. Usually 2. Not often 3. Not at all |
| 1. **A5** I get a sort of frightened feeling like “butterflies” in the stomach:   0. Not at all 1. Occasionally 2. Quite often 3. Very often |
| 1. **A6** I feel restless as I have to be on the move:   3. Very much indeed 2. Quite a lot 1. Not very much 0. Not at all |
| 1. **A7** I get sudden feelings of panic:   3. Very often indeed 2. Quite often 1. Not very often 0. Not at all |
| 1. **D1** I still enjoy the things I used to enjoy:   0. Definitely as much 1. Not quite so much  2. Only a little 3. Hardly at all |
| 1. **D2** I can laugh and see the funny side of things:   0. As much as I always could 1. Not quite so much now  2. Definitely not so much now 3. Not at all |
| 1. **D3** I feel cheerful   3. Not at all 2. Not often 1. Sometimes 0. Most of the time |
| 1. **D4** I feel as if I am slowed down:   3. Nearly all the time 2. Very often 1. Sometimes 0. Not at all |
| 1. **D5** I have lost interest in my appearance:   3. Definitely  2. I don´t take as much care as I should  1. I may not take quite as much care  0. I take just as much care as ever |
| 1. **D6** I look forward to enjoyment to things:   0. As much as I ever did 1. Rather less than I used to  2. Definitely less than I used to 3. Hardly at all |
| 1. **D7** I can enjoy a good book or radio or TV program:   0. Often 1. Sometimes 2. Not often 3. Very seldom |

Thank you very much for your participation,

The Medical Oncology team of the Hospital Universitario de La Princesa.
